# Low-Complexity Adaptive Sampling of Block Compressed Sensing Based on Distortion Minimization

**DOI:** 10.3390/s22134806

**Published:** 2022-06-25

**Authors:** Qunlin Chen, Derong Chen, Jiulu Gong

**Affiliations:** School of Mechatronical Engineering, Beijing Institute of Technology, Beijing 100081, China; 3120170115@bit.edu.cn (Q.C.); cdrmy@263.net (D.C.)

**Keywords:** adaptive sampling, block compressed sensing, distortion model, distortion minimization, image sampling, neural network

## Abstract

Block compressed sensing (BCS) is suitable for image sampling and compression in resource-constrained applications. Adaptive sampling methods can effectively improve the rate-distortion performance of BCS. However, adaptive sampling methods bring high computational complexity to the encoder, which loses the superiority of BCS. In this paper, we focus on improving the adaptive sampling performance at the cost of low computational complexity. Firstly, we analyze the additional computational complexity of the existing adaptive sampling methods for BCS. Secondly, the adaptive sampling problem of BCS is modeled as a distortion minimization problem. We present three distortion models to reveal the relationship between block sampling rate and block distortion and use a simple neural network to predict the model parameters from several measurements. Finally, a fast estimation method is proposed to allocate block sampling rates based on distortion minimization. The results demonstrate that the proposed estimation method of block sampling rates is effective. Two of the three proposed distortion models can make the proposed estimation method have better performance than the existing adaptive sampling methods of BCS. Compared with the calculation of BCS at the sampling rate of 0.1, the additional calculation of the proposed adaptive sampling method is less than 1.9%.

## 1. Introduction

Compressed sensing (CS) acquires the measurements of a signal by linear projection [1,2,3,4,5]. When CS is applied to image/video data, block compressed sensing (BCS) [6] is usually used to avoid the enormous calculation and storage pressure caused by the large-scale measurement matrix. In BCS, an image is divided into non-overlapping blocks of size *B* × *B*. The block size *B* is usually much smaller than the width or height of the image. The same measurement matrix measures the raster scan vector of each block. Since different blocks contain different amounts of valuable information, adaptive block compressed sensing (ABCS) methods [7,8,9,10,11,12,13,14,15,16] have been proposed to make full use of limited measurement resources.

In CS theory, the number of measurements required to recover a signal with high probability is proportional to its sparsity [4]. Therefore, the ABCS methods usually adaptively allocate the sampling rate of each image block by using the features that directly or indirectly reflect the sparsity of the image block. These features are often referred to as allocation factors [7]. According to the source of information, the allocation factors of the ABCS schemes include the transform domain features, spatial domain features, and measurement domain features.

In transform domain features, the allocation factors are extracted from the transformation coefficients of the image. Yu et al. [8] proposed a saliency feature based on Pulsed cosine transform (PCT), which is used to allocate fewer sensing resources to non-salient regions and more to salient regions. Zhu et al. [7] used the statistical information of image blocks in the transform domain as the allocation factors for allocating sampling rates of blocks. These allocation factors include the entropy of DCT coefficients, the variation of DCT coefficients, and the number of significant DCT coefficients. Zhang et al. [15] divided all image blocks into three categories based on the DCT coefficients of decoded images and allocated different sampling rates to each category. These methods require performing a two-dimensional (2D) transform of the image, which will bring additional computational complexity to the encoder.

To avoid the complexity of image transformation, some researchers used spatial domain features of the image block as the allocation factor. Wang et al. [17] and Luo et al. [18] used the variance and standard deviation as the allocation factor. Zhang et al. [19] used the standard deviations of blocks as the allocation factor. Many other image features are also effective for ABCS, such as the spatial entropy of blocks [20], the error between blocks [21], the block-based gradient field [11], the block boundary variation [12], and the statistical texture distinctiveness [9]. Moreover, some researchers combined multiple features to allocate the sampling rate of each block. Heng et al. [16] used fuzzy logic systems to select allocation factors from saliency and standard deviation features. Xu et al. [14] took the human visual system characteristics and signal sparsity as allocation factors. Nevertheless, if the original image is unavailable, the spatial and transform domain features cannot be used for the ABCS, such as compressed imaging (CI) architectures [22].

To avoid extracting features from the original image, some researchers extracted the features of CS measurements as the allocation factors. Li et al. [10] took the measurement contrast (MC) between the CS measurements of blocks as an allocation factor for allocating the block sampling rates. Li et al. [13] designed an allocation factor called sensing entropy, which estimates the statistical information of block pixel values from CS measurements. However, most allocation factors have high computational complexity, which cannot guarantee the simplicity of the CS encoder.

Based on the allocation factors, there are usually iterative [13,14] and non-iterative [7,8,9,10,11,12,17,18,19,20,21] methods used to allocate the sampling rate of each block. Since the non-iterative method has low complexity, it is suitable for the BCS encoder. However, the existing non-iterative methods of the ABCS schemes usually use the category or proportion of allocation factors to allocate the block sampling rates, resulting in a limited improvement of the rate-distortion performance.

In this paper, we propose a low-complexity ABCS scheme. The allocation problem of block sampling rates is transformed into a distortion minimization problem to pursue a better performance of the ABCS scheme. Three simple distortion models are used to describe the relationship between the block distortion and the sampling rates. The parameters of the distortion models are used as allocation factors, which are predicted by a simple neural network. Based on distortion minimization, a fast estimation method of block sampling rate is proposed.

The rest of the paper is organized as follows. Section 2 mainly analyzes the additional computational complexity of the existing ABCS schemes. The proposed ABCS scheme is given in Section 3. In Section 4, the proposed distortion model of the image block is discussed. The fast estimation method of the block sampling rate is given in Section 5. The simulation results are described in Section 6, and some conclusions are drawn in Section 7.

## 2. Proposed Scheme

Let $x_{i}\in {\mathbb{R}}^{B^{2}\times 1}(i=1,2,\cdots ,N)$ denote the column vector of the *i*th block in an image. The measurement vector $y_{i}\in {\mathbb{R}}^{M_{i}\times 1}$ of $x_{i}$ can be obtained by

(1) $$y_{i}=\Phi_{M_{i}}x_{i},$$

where $\Phi_{M_{i}}\in {\mathbb{R}}^{M_{i}\times B^{2}}$ represents the measurement matrix that should satisfy the restricted isometry property (RIP) [1,4], $M_{i}$ represents the number of measurements of the *i*th block, and $m_{i}=M_{i}/B^{2}$ is the sampling rate of the *i*th block. In BCS, all blocks have the same sampling rate, while in the ABCS schemes, all blocks may have different sampling rates. Compared with the BCS, the ABCS schemes can obtain more valuable information to improve the quality of the reconstructed images.

The purpose of the adaptive sampling method of BCS is to improve the quality of the reconstructed image as much as possible by using limited measurement resources. Therefore, the problem of allocating the sampling rates of blocks can be modeled as a distortion minimization problem, which can be expressed as

(2) $$\begin{array}{l} \begin{array}{cc} \underset{m_{1},m_{2},\ldots ,m_{N}}{\arg\min} & D(m_{1},m_{2},\ldots ,m_{N}) \end{array}\\ \begin{array}{cc} s.t. & \{\begin{array}{l} \sum_{i=1}^{N}m_{i}\leq Nm_{\mathrm{goal}}\\ m_{l}\leq m_{i}\leq m_{u},i=1,\ldots ,N \end{array} \end{array} \end{array}$$

where $m_{l}$ represents the lower bound of the block sampling rate, $m_{u}$ is the upper bound of block sampling rate, *N* represents the number of image blocks, and $D(m_{1},m_{2},\ldots ,m_{N})$ represents the distortion for the image at block sampling rates of $m_{1}$, $m_{2}$, …, and $m_{N}$.

**Figure 1:**
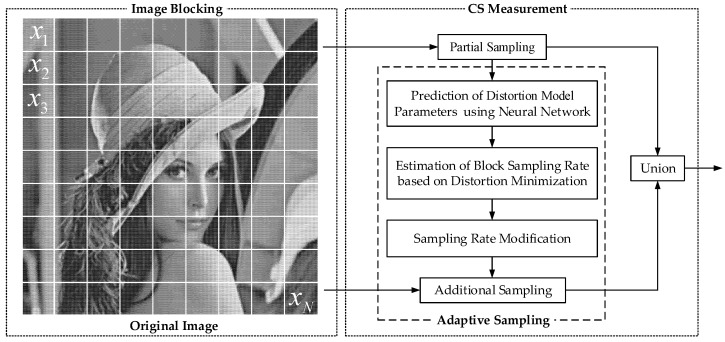
The proposed ABCS scheme.

To solve the problem (2) with low complexity, we use three simple functions to model the relationship between distortion and block sampling rate. The model parameters are used as the allocation factors and will be extracted by a simple neural network. We use the two-stage method to solve the problem (2). The analytical solution of the sampling rate is solved by minimizing the distortion problem without inequality constraints. Then, the analytical solution is modified by the inequality constraints to quickly estimate the sampling rates. The proposed ABCS scheme is shown in Figure 1. Firstly, an image is divided into non-overlapping blocks. Each block is measured by the partial sampling to obtain $M_{0}$ CS measurements. Secondly, the neural network extracts feature from the $M_{0}$ CS measurements to predict model parameters. Then, the number of measurements $M_{i}$ of the *i*th block is estimated based on the proposed estimation method. Finally, $M_{i}-M_{0}$ additional CS measurements of the *i*th block are sampled by the additional sampling, and these samples are united with the $M_{0}$ CS measurements as the final measurements of the block.

## 3. Complexity Analysis of the Existing ABCS Schemes

The existing adaptive sampling methods of BCS usually include two steps. The first step is to extract the allocation factor. The allocation factor $T_{i}$ of the *i*th block can be expressed as follows:

(3) $$T_{i}=f(X_{i}),$$

where $X_{i}\in {\mathbb{R}}^{B\times B}$ represents the matrix of the *i*th block and $f:{\mathbb{R}}^{B\times B}\to \mathbb{R}$ represents the operations of feature extraction. The second step is to calculate the sampling rate or the number of measurements of each block according to the allocation method. The most commonly used non-iterative allocation method [7,8,10,11,12,13,14,15,16,19] uses the linear proportion of the allocation factor to estimate the sampling rates. The sampling rate $m_{i}$ of the *i*th block can be expressed as follows:

(4) $$m_{i}=m_{l}+\left(T_{i}/\sum_{i=1}^{N}T_{i}\right)N\left(m_{\mathrm{goal}}-m_{l}\right),$$

where $m_{l}$ represents the lower bound of the block sampling rate, $m_{\mathrm{goal}}$ represents the given sampling rate of the image, and *N* represents the number of image blocks.

Low complexity is the reason for using BCS, so the ABCS scheme should first consider the additional computational complexity caused by adaptive sampling. In Equation (4), $\sum_{i=1}^{N}T_{i}$ only needs to calculate once for all blocks. Each block needs two multiplications, one division, two additions, and one subtraction on average to calculate Equation (4). Compared with BCS, the additional computational complexity of the ABCS schemes with non-iterative allocation methods mainly comes from allocation factors. We analyze the computational complexity of the features commonly used in allocation factors. Table 1 shows the number of basic operations for the features of the block $X_{i}$.

Since multiplication is much more complicated than addition, the number of multiplications is used to measure the additional computational complexity of the allocation factors in this study. In Table 1, $X_{d}\in {\mathbb{R}}^{I_{d}\times I_{d}}$ is the down-sampled image of the original image $X\in {\mathbb{R}}^{I\times I}$. The down-sampled image size $I_{d}\times I_{d}$ affects both the complexity of PCT and the block sparsity measured by PCT.

Supposing that the image size I×I is 256 × 256, the block size B×B is 16 × 16, and the down-sampled image size $I_{d}\times I_{d}$ is 64 × 64. The CS sampling of a block with a sampling rate of 0.1 requires $M_{i}B^{2}$= 6656 multiplications. The 2D transform, PCT, variance, MC, and sensing entropy require 8192, 5264, 258, 19,661, and 2820 multiplications, respectively. The multiplication calculations of 2D transform, PCT, variance, MC, and sensing entropy account for 123.08%, 79.09%, 3.88%, 295.39%, and 42.37% of the multiplication calculation of the CS measurements, respectively.

The transform domain features need to transform the image block by a 2D transform, so their multiplication calculations are larger than 8192. Since the calculation of the probability $p_{j}$ and $\log(p_{j})$ have high computational complexity, the computational complexity of the spatial entropy is larger than that of the variance for a block.

Based on the above discussion, the computational complexity of block variance is the lowest among the existing allocation factors. Nevertheless, the variance cannot be used in applications where the original image is unavailable. The measurement domain features do not need the original image or image transformation, but the current measurement domain features have high computational complexity.

In the ABCS schemes based on the measurement domain features, the allocation factor is first extracted from $M_{0}$ CS measurements. After the number of measurements $M_{i}$ of the *i*th block is allocated, the encoder makes a judgment for additional sampling. The encoder acquires $M_{i}-M_{0}$ additional measurements if $M_{i}$ is greater than $M_{0}$. The encoder discards $M_{0}-M_{i}$ measurements if $M_{i}$ is less than $M_{0}$. Therefore, the total number of additional multiplications caused by a measurement domain feature is $M_{0}L+\max\left\{0,(M_{0}-M_{i})\right\}B^{2}$, where *L* represents the average number of multiplications required per measurement for calculating the measurement domain feature.

In order to make the computational complexity of the measurement domain feature lower than the variance, it is necessary to satisfy the following condition:

(5) $$M_{0}L+\max\left\{0,(M_{0}-M_{i})\right\}\leq B^{2}+2.$$

From Equation (5), we can derive

(6) $$\{\begin{array}{l} M_{0}\leq M_{i}\\ L\leq \left(B^{2}+2\right)/M_{0} \end{array}.$$

It can be seen from Equation (6) that $M_{0}$ should be as small as possible, and the number of multiplications for extracting features cannot exceed $\left(B^{2}+2\right)/M_{0}$. In this study, the $M_{0}$ CS measurements are used as the input of the neural network to extract the allocation factors. By setting the number of neurons in the input and hidden layers of the neural network, the additional computational complexity of the proposed ABCS scheme can easily satisfy the conditions in Equation (6).

## 4. Block Distortion Model of BCS

### 4.1. Sampling Rate and Distortion for Image Block

According to CS theory, the sampling rate has a decisive impact on the distortion of the reconstructed image. We use the mean absolute difference (MAD) between the original and reconstructed image blocks to measure the distortion [23]. The MAD of the *i*th reconstructed image block can be expressed as

(7) $$D_{i}=\frac{1}{B^{2}}\sum_{j}\sum_{k}\left|X_{i}(j,k)-{\hat{X}}_{i}(j,k)\right|,$$

where $X_{i}(j,k)$ and ${\hat{X}}_{i}(j,k)$ represent the pixel values of the *i*th original and reconstructed image blocks, respectively.

**Figure 2:**
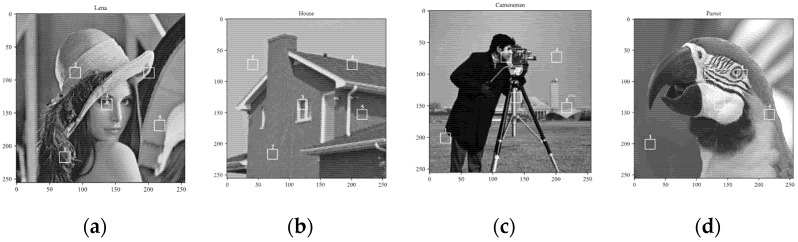

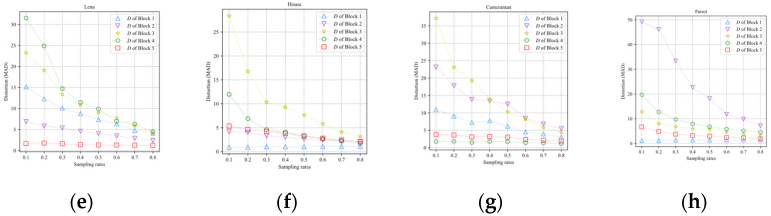
The MAD curves of different blocks: (**a**) Lena; (**b**) House; (**c**) Cameraman; (**d**) Parrot. (**e**) The MAD curves of five image blocks in Lena. (**f**) The MAD curves of five image blocks in House. (**g**) The MAD curves of five image blocks in Cameraman. (**h**) The MAD curves of five image blocks in Parrot.

In general, the distortion of the reconstructed image decreases with the increase in the sampling rate. Our experiment on several image blocks analyzes the relationship between distortion and sampling rate. Figure 2 shows the MAD curves of different blocks in Lena, House, Cameraman, and Parrot. It can be found that with the increase in the sampling rate, the MAD and the declining rate of MAD decrease gradually. The main reason for this phenomenon is that the redundant information of the measurements increases with the number of measurements.

In addition, the block distortion curve is related to the content of the image block. The MAD of smooth image blocks decreases slowly with the increase in the sampling rate. For example, Block 5 of Lena, Block 1 of House, Block 4 of Cameraman, and Block 1 of Parrot belong to smooth blocks. The distortion curves of these blocks are approximately horizontal lines, and the gradient of the curve is almost zero. On the contrary, the MAD of complex image blocks decreases rapidly with the increase in the sampling rate. Block 4 of Lena, Block 3 of House, Block 3 of Cameraman, and Block 2 of Parrot belong to complex blocks. The declining rate of the MAD curve of these blocks is much faster than that of smooth blocks.

### 4.2. Distortion Model of Image Block for BCS

In order to quickly solve the problem (2), we model the block distortion $D_{i}$ as a continuous convex function of the sampling rate $m_{i}$. According to the characteristics of curves in Figure 2, $D_{i}$ needs to satisfy the following conditions:

(8) $$\{\begin{array}{c} \frac{\partial D_{i}}{\partial m_{i}}\leq 0\\ \frac{\partial^{2}D_{i}}{\partial m_{i}^{2}}\geq 0 \end{array}.$$

To ensure the low complexity for predicting distortion, we use the exponential function, quadratic polynomial function, and logarithmic function to establish the following three models between $D_{i}$ and $m_{i}$ for the *i*th block:

(9) $$D_{i}(m_{i})=a_{i,1}2^{a_{i,2}m_{i}}+a_{i,3},$$

(10) $$D_{i}(m_{i})=b_{i,1}{\left(m_{i}+b_{i,2}\right)}^{2}+b_{i,3},$$

(11) $$D_{i}(m_{i})=c_{i,1}\ln(m_{i}+c_{i,2})+c_{i,3}.$$

**Figure 3:**
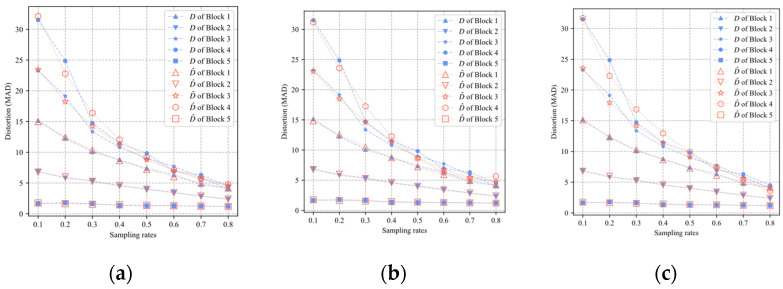
The predicted distortion $\hat{D}$ of five blocks of Lena for different models: (**a**) The exponential model; (**b**) the polynomial model; (**c**) the logarithmic model.

In Equations (9)–(11), $a_{i,1}$, $a_{i,2}$, $a_{i,3}$, $b_{i,1}$, $b_{i,2}$, $b_{i,3}$, $c_{i,1}$, $c_{i,2}$, and $c_{i,3}$ are the parameters related to the content of the *i*th block. The index of the block will be omitted and the parameters of the models also be expressed by $a_{1}$, $a_{2}$, $a_{3}$, $b_{1}$, $b_{2}$, $b_{3}$, $c_{1}$, $c_{2}$, and $c_{3}$. According to Equation (8), it can be concluded that $a_{i,1}\geq 0$, $a_{i,2}\leq 0$, $b_{i,1}\geq 0$, $b_{i,2}\leq -1$, $c_{i,1}\leq 0$, and $c_{i,2}\geq 0$. The three models (9)–(11) are tested on the five blocks of Figure 2a. Firstly, each block is measured and reconstructed by using BCS and the smoothed projected Landweber (SPL) algorithm based on DCT [24], respectively, where the sampling rate set is {0.1, 0.2, …, 0.8}. The block distortion D is calculated by the original image block and the reconstructed image block. There are eight samples of distortion for each block. Then, the least square fitting method (LSF) is used to fit eight samples of the distortion to obtain the model parameters of three models for the block. Finally, the block distortion models are used to predict the distortion at different sampling rates. The predicted distortion is recorded as $\hat{D}$. The distortion D and the predicted distortion $\hat{D}$ of the five blocks of Lena are shown in Figure 3. It can be seen that the three distortion models can predict the distortion of the reconstruction image accurately.

**Figure 4:**
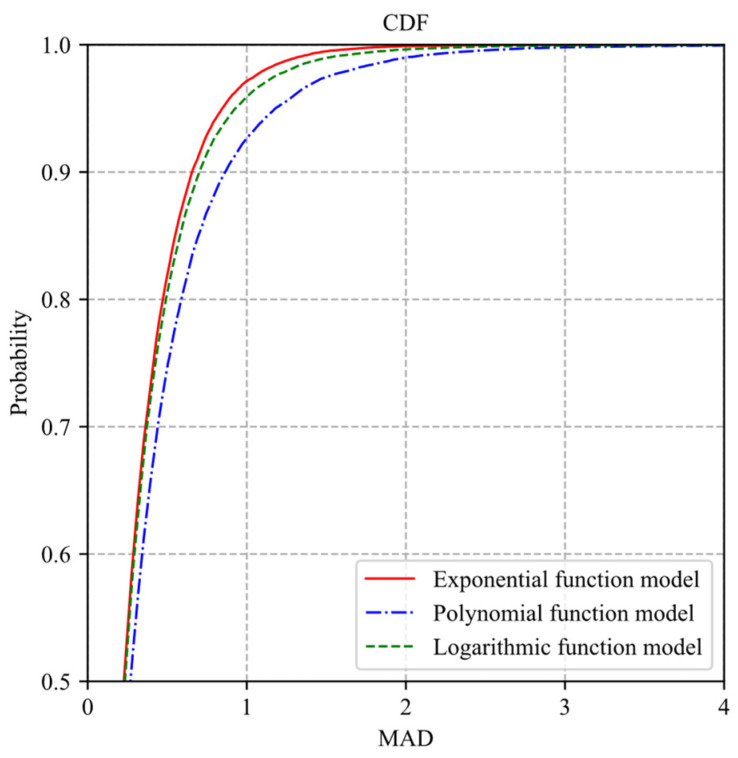
CDF curves of MADs of prediction distortion vectors predicted by three distortion models.

The three models are also tested on 25,600 blocks in 100 images of the BSDS500 dataset [25]. The distortion model of each block is obtained by fitting eight sample pairs of sampling rate-distortion, where the sampling rate set is {0.1, 0.2, …, 0.8}. The distortion vector and the prediction distortion vector of each block are composed of eight distortions and eight prediction distortions, respectively. The MAD between the block distortion vector and the prediction distortion vector is used to evaluate the fitting performance of distortion models. We calculate the MAD between the distortion vector and the predicted distortion vector for the 25,600 blocks. The cumulative distribution function (CDF) curves of the MADs of the prediction distortion vectors predicted by the three distortion models are shown in Figure 4. The MADs of prediction distortion vectors of the three models are almost less than 4. The MADs of prediction distortion vectors of the three models are less than 2 for 99% of the 25,600 samples. These show that the three models can effectively describe the correlation between sampling rate and distortion for each block. Among the three models, the fitting performances of the exponential model, logarithmic model, and polynomial model decrease in turn.

## 5. Adaptive Sampling of BCS Based on Distortion Minimization

The MAD of an image is the average MAD of all image blocks according to Equation (7). As the objective function, the sum of MADs of all blocks has the same effect as the average of MADs. Therefore, the problem of allocating the block sampling rates can be expressed as

(12) $$\begin{array}{l} \begin{array}{cc} \underset{m_{1},m_{2},\ldots ,m_{N}}{\arg\min} & \sum_{i=1}^{N}D_{i}(m_{i}) \end{array}\\ \begin{array}{cc} s.t. & \{\begin{array}{l} \sum_{i=1}^{N}m_{i}\leq Nm_{\mathrm{goal}}\\ m_{l}\leq m_{i}\leq m_{u},i=1,\ldots ,N \end{array} \end{array} \end{array}$$

Equation (12) can be converted to an unconstrained problem, which can be expressed as

(13) $$\begin{array}{cc} \underset{m_{1},m_{2},\ldots ,m_{N}}{\arg\min} & F=\sum_{i=1}^{N}D_{i}+\lambda \left(\sum_{i=1}^{N}m_{i}-Nm_{\mathrm{goal}}\right) \end{array}+\sum_{i=1}^{N}u_{i}(m_{i}-m_{u})+\sum_{i=1}^{N}v_{i}(m_{l}-m_{i}),$$

where λ, $u_{i}$, and $v_{i}$ are the Lagrangian multipliers. Since the dimension of the unknown variable of the above problem is 3N+1, it is not easy to obtain an analytical solution for the sampling rates. Similar inequality constraint problems are usually solved iteratively by the reverse water-filling idea [26], but this method has high computational complexity and is unsuitable for BCS.

To optimize problem (12) with low complexity, we use two stages to estimate the sampling rates. In the first stage, the analytical solution of the sampling rate is solved by minimizing the distortion problem without inequality constraints. In the second stage, the analytical solution is modified by the inequality constraints to quickly estimate the sampling rates.

### 5.1. Analytical Solution of Block Sampling Rates

In general, the optimal block sampling rates should make full use of the given sampling rate of the image; that is,

(14) $$\sum_{i=1}^{N}m_{i}=Nm_{\mathrm{goal}}.$$

Without the inequality constraints, we can express the allocation problem of the block sampling rates as

(15) $$\begin{array}{l} \begin{array}{cc} \underset{m_{1},m_{2},\ldots ,m_{N}}{\arg\min} & D=\sum_{i=1}^{N}D_{i} \end{array}\\ \begin{array}{cc} s.t. & \sum_{i=1}^{N}m_{i}=Nm_{\mathrm{goal}} \end{array} \end{array}$$

Equation (15) can be converted to an unconstrained optimization problem, which can be expressed as

(16) $$\begin{array}{cc} \underset{m_{1},m_{2},\ldots ,m_{N}}{\arg\min} & F(m_{1},m_{2},\ldots ,m_{N})=\sum_{i=1}^{N}D_{i}+\lambda \left(\sum_{i=1}^{N}m_{i}-Nm_{\mathrm{goal}}\right) \end{array}.$$

Let $\frac{\partial F(m_{1},m_{2},\ldots ,m_{N})}{\partial m_{i}}=0$, we can derive

(17) $$\frac{\partial D_{i}}{\partial m_{i}}=-\lambda .$$

According to a model of distortion, we can quickly estimate the analytical solution of the sampling rates by combining Equations (14) and (17).

#### 5.1.1. Exponential Model

When using the exponential model (9) to describe the relationship between distortion and sampling rate, Equation (17) can be expressed as

(18) $$a_{i,1}a_{i,2}2^{a_{i,2}m_{i}}=-\lambda .$$

From Equation (18), the sampling rate of the *i*th block can be expressed as

(19) $$m_{i}=\frac{1}{a_{i,2}}\log_{2}\frac{-\lambda }{a_{i,1}a_{i,2}}=\frac{1}{a_{i,2}}\left(\log_{2}(\lambda )-\log_{2}(-a_{i,1}a_{i,2})\right).$$

By substituting Equation (19) into Equation (14), we can obtain

(20) $$\log_{2}(\lambda )=\left(Nm_{\mathrm{goal}}-\sum_{i=1}^{N}\left(\frac{1}{a_{i,2}}\log_{2}\frac{-1}{a_{i,1}a_{i,2}}\right)\right)/\sum_{i=1}^{N}\frac{1}{a_{i,2}}.$$

The block sampling rate of the *i*th block can be obtained by substituting Equation (20) into Equation (19).

#### 5.1.2. Polynomial Model

When using the polynomial model (10) to describe the relationship between distortion and sampling rate, Equation (17) can be expressed as

(21) $$2b_{i,1}(m_{i}+b_{i,2})=-\lambda .$$

From Equation (21), the sampling rate of the *i*th block can be expressed as

(22) $$m_{i}=-b_{i,2}-\frac{\lambda }{2b_{i,1}}.$$

By substituting Equation (22) into Equation (14), we can obtain

(23) $$\lambda =\left(-\sum_{i=1}^{N}b_{i,2}-Nm_{\mathrm{goal}}\right)/\sum_{i=1}^{N}\frac{1}{2b_{i,1}}.$$

The block sampling rate of the *i*th block can be obtained by substituting Equation (23) into Equation (22).

#### 5.1.3. Logarithmic Model

When using the logarithmic model (11) to describe the relationship between distortion and sampling rate, Equation (17) can be expressed as

(24) $$\frac{c_{i,1}}{m_{i}+c_{i,2}}=-\lambda .$$

From Equation (24), the sampling rate of the *i*th block can be expressed as

(25) $$m_{i}=\frac{c_{i,1}}{-\lambda }-c_{i,2}.$$

By substituting Equation (25) into Equation (14), we can derive

(26) $$\lambda =\frac{\sum_{i=1}^{N}c_{i,1}}{-\sum_{i=1}^{N}c_{i,2}-Nm_{\mathrm{goal}}}.$$

The block sampling rate of the *i*th block can be obtained by substituting Equation (26) into Equation (25).

### 5.2. Sampling Rate Modification

To ensure that the analytical solution of the sampling rate meets the upper and lower bounds, the sampling rate of the *i*th block is modified as follows:

(27) $$m_{i}^{\left(1\right)}=\{\begin{array}{ll} m_{l} & m_{i}<m_{l}\\ m_{i} & m_{l}\leq m_{i}\leq m_{u}\\ m_{u} & m_{i}>m_{u} \end{array}.$$

The block sampling rates modified by Equation (27) do not meet Equation (14), so we adjust the block sampling rates as

(28) $$m_{i}^{\left(2\right)}=\{\begin{array}{ll} m_{i}^{\left(1\right)}+\frac{m_{\mathrm{goal}}-\sum_{i=1}^{N}m_{i}^{\left(1\right)}}{N} & \sum_{i=1}^{N}m_{i}^{\left(1\right)}<Nm_{\mathrm{goal}}\\ m_{l}+\frac{m_{i}^{\left(1\right)}-m_{l}}{\sum_{i=1}^{N}\left(m_{i}^{\left(1\right)}-m_{l}\right)}\left[N\left(m_{\mathrm{goal}}-m_{l}\right)\right] & \sum_{i=1}^{N}m_{i}^{\left(1\right)}\geq Nm_{\mathrm{goal}} \end{array}.$$

### 5.3. Model Parameters Prediction Based on Neural Network

According to Equations (19), (22) and (25), the parameters of the distortion models are key to allocating block sampling rates. Since the neural network [27] does not need to reveal the mapping relationship between variables in advance but only to ensure the correlation between variables, the neural network is suitable for predicting the model parameters.

The input characteristics are key to predicting the parameters of the block distortion model by a neural network. The distortion model parameters are related to the content of the image block. The CS measurements can reconstruct the image, which indicates that the CS measurements contain the feature information of the image. Therefore, several CS measurements can be used as neural network inputs to predict model parameters.

Using CS measurements to predict model parameters is a regression problem, which is suitable to be solved by a feed-forward neural network. The four-layer feed-forward neural network can give the input–target relations exactly with fewer hidden neurons than the three-layer feed-forward neural network [28]. Therefore, we use a four-layer feed-forward neural network to predict the model parameters.

**Figure 5:**
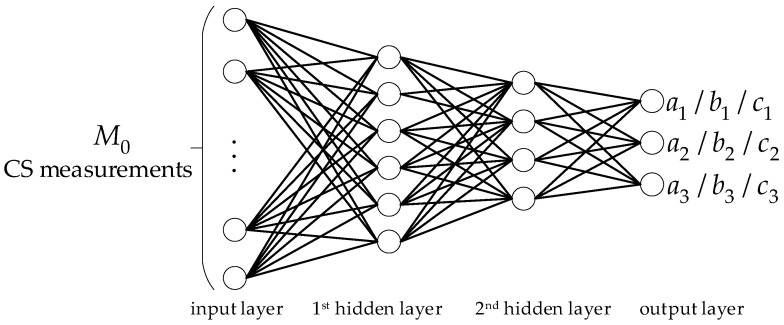
The neural network structure of predicting model parameters.

Based on Equation (6), the number of CS measurements used to predict the model parameters should be as small as possible. However, too few measurements cannot guarantee the effectiveness of the extracted features. In Refs. [29,30], some useful features were successfully extracted from thirteen CS measurements. If the number of input neurons is 13, the numbers of neurons in the two hidden layers are set to six and four, according to Ref. [28]. The designed neural network structure is shown in Figure 5. $M_{0}$ CS measurements are used as the input of the neural network. We discuss the influence of $M_{0}$ on reconstruction performance in Section 6.1. Due to the simple calculation, the ReLU function [31] is used as the activation function of two hidden layers. The mean square error is used as the loss function for training the neural network.

The multiplication required for predicting the model parameters used by the trained network is $M_{0}\times 6+6\times 4+4\times 2=6M_{0}+32$. If $M_{0}\leq \left(B^{2}-32\right)/6$, the computational complexity of predicting model parameters will not be higher than that of the image block variance.

Since the fitting intervals of the model parameters are continuous intervals, the fitted values of the model parameters have high diversity. The more diverse the parameters are, the more difficult it is for the neural network to predict the model parameters accurately. Therefore, we propose discretizing the fitting intervals of the model parameters as a finite value set.

#### 5.3.1. Parameter Discretization of the Exponential Model

**Figure 6:**
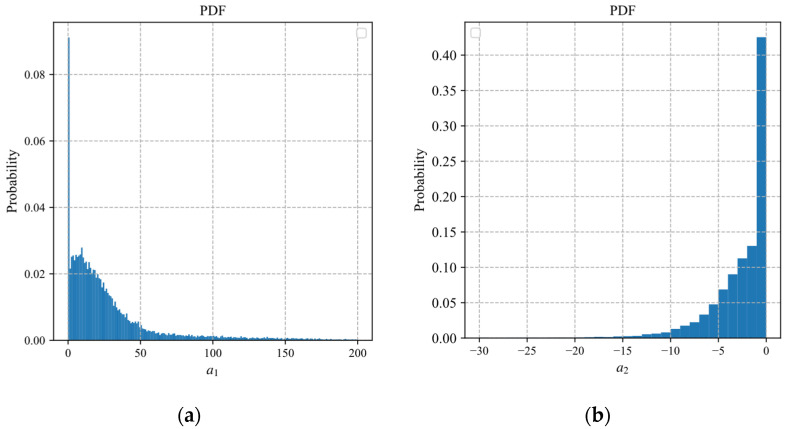
Probabilistic histograms of parameters: (**a**) $a_{1}$; (**b**) $a_{2}$.

According to Equation (19), the estimation of the sampling rate depends on the parameters $a_{1}$ and $a_{2}$ of the exponential model. Figure 6a,b displays probabilistic histograms of parameters $a_{1}$ and $a_{2}$ obtained by fitting the block distortion vectors of 25,600 blocks in 100 images of the BSDS500 dataset.

In Figure 6, the distribution of $a_{2}$ is more concentrated than that of $a_{1}$, where the values of $a_{2}$ are mainly concentrated between −10 and 0. To reduce the diversity of the fitted parameters with less influence, we discrete the fitting interval of the parameter $a_{2}$. The discretization form of the exponential distortion model of the *i*th block can be expressed as

(29) $$D_{i}(m_{i})=\sum_{k=1}^{K}\left(w_{k}\left(a_{k,i,1}2^{P_{k}m_{i}}+a_{k,i,3}\right)\right),$$

where $\sum_{k=1}^{K}w_{k}=1,\;w_{k}\in \{0,1\}$, $P_{k}\in (-\infty ,0)$ is a constant, $\left\{P_{k}\in (-\infty ,0),k=1,\ldots ,K\right\}$ represents the value set of $a_{2}$, and K is the number of the value set. The model (29) is equivalent to the original exponential model (9) at K→∞.

To analyze the influence of $a_{2}$ on the exponential model, we construct the vector function $G_{1}(a_{2})=[2^{0.1\times a_{2}},2^{0.2\times a_{2}},\ldots ,2^{0.8\times a_{2}}]$ with respect to $a_{2}$, where 0.1, 0.2, …, and 0.8 represent the most commonly used sampling rates. The integer values of the fitted interval of $a_{2}$ in Figure 6b are substituted into $G_{1}(a_{2})$. The Pearson correlation coefficients (PCC) between two different vectors of $G_{1}(a_{2})$ are shown in Table 2. It is observed that the vectors of $G_{1}(a_{2})$ have high correlation with each other. When the value difference of $a_{2}$ is 1, the PCCs between two different vectors of $G_{1}(a_{2})$ are greater than 0.998. For example, for the values −3 and −4, with a value difference of 1, the PCC is 0.998. Therefore, we assume $P_{k}\in \mathbb{Z}$ in the following discussion to quickly choose an optimal or near-optimal finite value set for $a_{2}$.

When the value set $\left\{P_{k}\in (-\infty ,0),k=1,\ldots ,K\right\}$ has only one element, the exponential distortion model (29) can be expressed as

(30) $$D_{i}(m_{i})=a_{i,1}2^{-P_{0}m_{i}}+a_{i,3}.$$

When the value set $\left\{P_{k}\in (-\infty ,0),k=1,\ldots ,K\right\}$ has only two elements, the exponential distortion model (29) can be expressed as

(31) $$D_{i}(m_{i})=w_{1}\left(a_{1,i,1}2^{P_{1}m_{i}}+a_{1,i,3}\right)+w_{2}\left(a_{2,i,1}2^{P_{2}m_{i}}+a_{2,i,3}\right).$$

**Figure 7:**
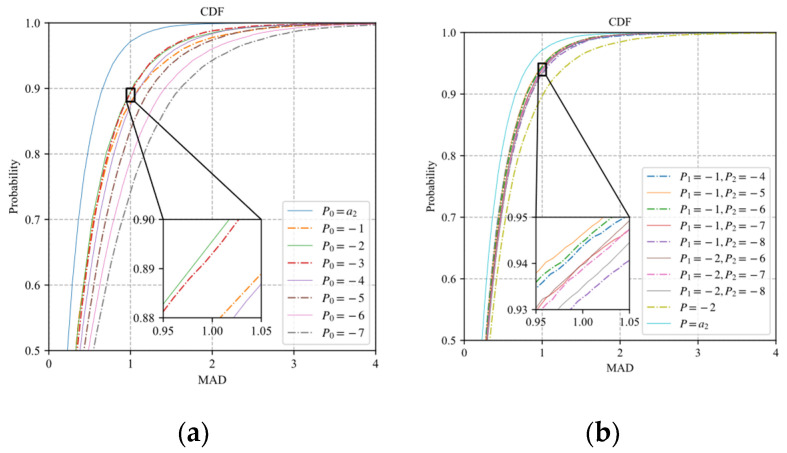
The CDF curves of MADs of the prediction distortion vectors: (**a**) model (30); (**b**) model (31).

Since the exponential distortion model can reduce the fitting error by adjusting $a_{1}$ and $a_{3}$, the optimal value set should minimize the fitting error rather than the discretization error. Similar to Figure 4, 25,600 samples of the block distortion vector are fitted by the model (30) and model (31) with different values of $P_{k}$. The CDF curves of MADs of the prediction distortion vectors are shown in Figure 7, where $P_{0}=a_{2}$ represents the results of the original exponential model without discretization. It is observed that the model (30) with $P_{0}=-2$ has the best fitting performance. The model (31) with $P_{1}=-1$ and $P_{2}=-5$ has the best fitting performance, which is close to that of the original exponential model. Therefore, the case that the number of $\left\{P_{k}\in (-\infty ,0),k=1,\ldots ,K\right\}$ is greater than two is not included in our discussion.

The discretization of the fitting interval of $a_{2}$ is used to collect the training samples of the model parameters. We fix $a_{2}$ as one value of the finite value set and then fit the model (9) by the distortion data to obtain the fitted values of the parameters $a_{1}$ and $a_{3}$ for each block. After traversing all the values of the finite set $\left\{P_{k}\in (-\infty ,0),k=1,\ldots ,K\right\}$, we can obtain K samples of the parameters $a_{1}$, $a_{2}$, and $a_{3}$ for each block. By comparing the fitted errors of the K samples, the parameters $a_{1}$, $a_{2}$, and $a_{3}$ with the least fitted error are used as the final training sample of the block. 

#### 5.3.2. Parameter Discretization of the Polynomial Model and the Logarithmic Model

**Figure 8:**
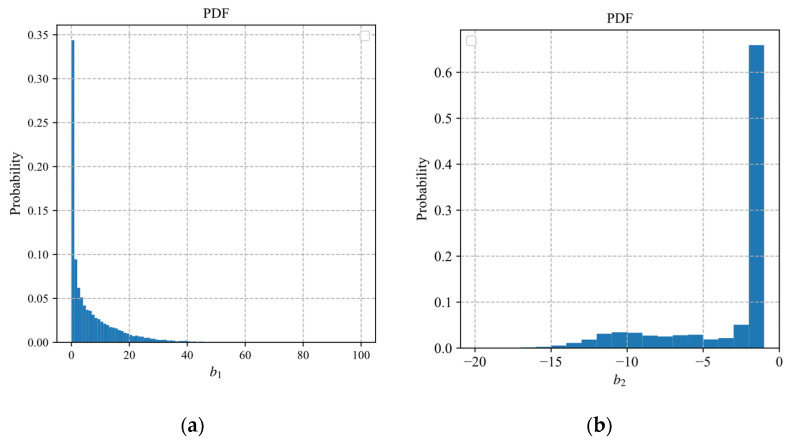
The probabilistic histograms of parameters $b_{1}$ and $b_{2}$: (**a**) $b_{1}$; (**b**) $b_{2}$.

**Figure 9:**
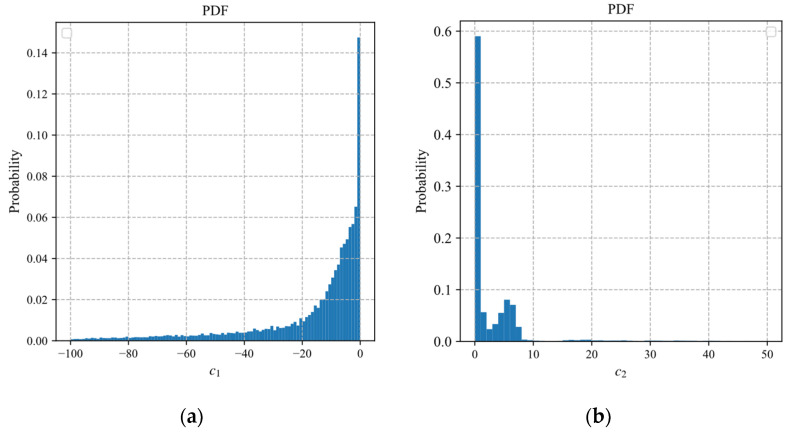
The probabilistic histograms of parameters $c_{1}$ and $c_{2}$: (**a**) $c_{1}$; (**b**) $c_{2}$.

In the same way as the exponential model, we discretize the parameters of the polynomial model and the logarithmic model. In the ABCS scheme based on the polynomial model (10), the estimation of the sampling rate depends on the parameters $b_{1}$ and $b_{2}$. In the ABCS scheme based on the logarithmic model (11), the estimation of the sampling rate depends on the parameters $c_{1}$ and $c_{2}$. We fit the 25,600 samples of the block distortion vector to obtain the fitted values of the parameters. The probabilistic histograms of parameters $b_{1}$ and $b_{2}$ are shown in Figure 8. The probabilistic histograms of parameters $c_{1}$ and $c_{2}$ are shown in Figure 9.

In Figure 8 and Figure 9, the distributions of $b_{2}$ and $c_{2}$ are more concentrated than those of $b_{1}$ and $c_{1}$. Similar to the parameter discretization of the exponential model, we discrete the fitting interval of the parameters $b_{2}$ and $c_{2}$. The discretization forms of the polynomial and logarithmic models can be expressed as

(32) $$D_{i}(m_{i})=\sum_{k=1}^{K}\left(w_{k}\left(b_{k,i,1}{\left(m_{i}+Q_{k}\right)}^{2}+b_{k,i,3}\right)\right),$$

(33) $$D_{i}(m_{i})=\sum_{k=1}^{K}\left(w_{k}\left(c_{k,i,1}\ln(m_{i}+T_{k})+c_{k,i,3}\right)\right),$$

where $\sum_{k}^{K}w_{k}=1,\;w_{k}\in \{0,1\}$, $Q_{k}\in (-\infty ,-1)$, and $T_{k}\in (0,+\infty )$ are constants; $\left\{Q_{k}\in (-\infty ,-1),k=1,\ldots ,K\right\}$ and $\left\{T_{k}\in (0,+\infty ),k=1,\ldots ,K\right\}$ represent the value sets of $b_{2}$ and $c_{2}$; and K is the number of the value set. The models (32) and (33) are equivalent to the original polynomial and logarithmic models at K→∞.

When K=1 or K=2, the polynomial distortion model (32) can be expressed as

(34) $$D_{i}(m_{i})=b_{i,1}{\left(m_{i}+Q_{0}\right)}^{2}+b_{i,3},$$

(35) $$D_{i}(m_{i})=w_{1}\left(b_{1,i,1}{\left(m_{i}+Q_{1}\right)}^{2}+b_{1,i,3}\right)+w_{2}\left(b_{2,i,1}{\left(m_{k}+Q_{2}\right)}^{2}+b_{2,i,3}\right).$$

When K=1 or K=2, the logarithmic distortion model (33) can be expressed as

(36) $$D_{i}(m_{i})=c_{i,1}\ln(m_{i}+T_{0})+c_{i,3},$$

(37) $$D_{i}(m_{i})=w_{1}\left(c_{1,i,1}\ln(m_{i}+T_{1})+c_{1,i,3}\right)+w_{2}\left(c_{2,i,1}\ln(m_{i}+T_{2})+c_{2,i,3}\right),$$

**Figure 10:**
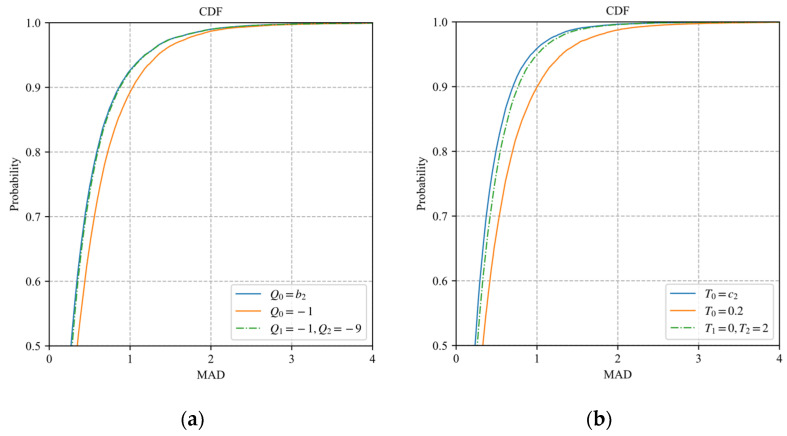
The CDF curves of MAD of the prediction distortion vectors predicted by models (34)–(37): (**a**) models (34)–(35); (**b**) models (36)–(37).

Similar to the Section 5.3.1, we obtain $Q_{0}=-1$, $Q_{1}=-1$, $Q_{2}=-9$, $T_{0}=0.2$, $T_{1}=0$, and $T_{2}=2$ for the model (34)–(37). Figure 10a shows the CDF curves of MADs of the prediction distortion vectors predicted by the different discretization forms of the polynomial model, where $Q_{0}=b_{2}$ represents the results of the original polynomial model without discretization. Figure 10b shows the CDF curves of MADs of the prediction distortion vectors predicted by the different discretization forms of the logarithmic model, where $T_{0}=c_{2}$ represents the results of the original logarithmic model without discretization. It is observed that the fitting performance of the discretized models is very close to the original model for the polynomial and logarithmic models. The main reason is that the value ranges of the parameters $b_{2}$ and $c_{2}$ are concentrated in a finite range. After $b_{2}$ and $c_{2}$ are fixed as a value, the models can reduce the fitting error by adjusting the other two parameters.

### 5.4. The Proposed Adaptive Sampling Method of BCS

In this study, we allocate the block sampling rates based on distortion minimization. We propose three functional models to describe the relationship between block sampling rate and distortion. Any of the three models can be used to estimate the block sampling rate. The parameters of each model are predicted by the neural network, in which the neural network is trained off-line on a large dataset. In practical application, the model parameters of each block can be predicted according to the trained neural network, and the sampling rate of each block can be quickly estimated by the proposed formula. The process of adaptive sampling is as follows:

(1) Partial sampling

Each block is measured to obtain $M_{0}$ CS measurements, where the sampling rate is $m_{0}$.

(2) Model parameters prediction

The parameters of the distortion model are predicted by the trained neural network, where the *M*_0_ CS measurements are used as the input of the trained neural network.

(3) Block sampling rate estimation

The block sampling rate is calculated according to Equation (19), Equation (22), or Equation (25).

(4) Sampling rate modification

The estimated sampling rates are modified by Equations (27) and (28). To avoid the significant difference in the number of measurements between blocks, we take the lower bound $m_{l}=0.4m_{\mathrm{goal}}$. In general, the upper bound $m_{u}=0.8$.

(5) Additional sampling

The *i*th block is measured to obtain $M_{i}-M_{0}$ additional measurements, where the sampling rate is $m_{i}^{\left(2\right)}-m_{0}$. The $M_{i}-M_{0}$ additional measurements are united with the $M_{0}$ measurements as the final measurements of the *i*th block.

## 6. Experimental Results and Discussion

**Figure 11:**
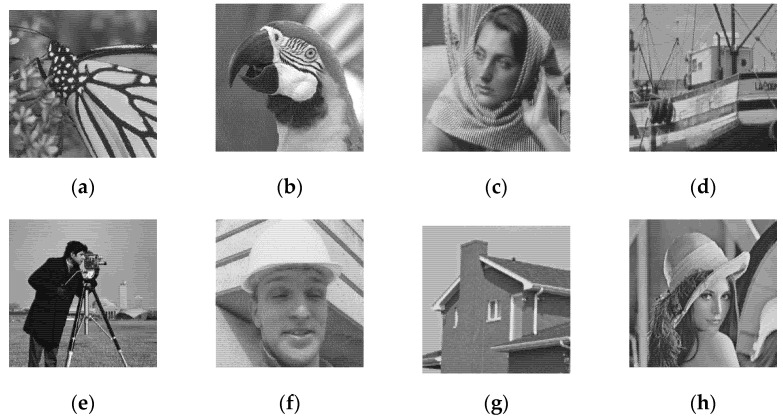
Eight test images: (**a**) Monarch; (**b**) Parrot; (**c**) Barbara; (**d**) Boats; (**e**) Cameraman; (**f**) Foreman; (**g**) House; (**h**) Lena.

The proposed adaptive sampling schemes are tested on different images, which include Monarch, Parrot, Barbara, Boats, Cameraman, Foreman, House, and Lena, as shown in Figure 11 [32], and 68 images from the BSD68 dataset [33]. One hundred images of the BSDS500 dataset [25] are used to collect samples of the training set. The images of the BSD68 and BSDS500 datasets are clipped to a size of 256 × 256 in the center. In the experimental test, the set of sampling rate is {0.1, 0.2, 0.3, 0.4, 0.5}. The encoder and decoder algorithms all use the same configuration except for the sampling method. The same random Gaussian matrix is used at the encoder to measure the image block. The image block size is 16 × 16, and the CS measurements are uniformly quantized with 8 bits. The decoder uses BCS-SPL-DCT [24] to reconstruct the image. All numerical experiments are simulated by Python and Octave on a computer equipped with a Windows 10 (64-bit) system and Intel Core i5-8300H 2.30 GHz processor and 16 GB RAM.

The proposed ABCS schemes need to train the neural network in advance. Firstly, 25,600 blocks of 100 images in the BSDS500 dataset are used to collect the block distortions at different sampling rates, where the set of sampling rates is {0.1, 0.2, …, 0.8}. Then, the least square fitting method (LSF) is used to fit the block distortions to obtain the model parameters for each block. If the fitting error is less than five, $M_{0}$ CS measurements and the fitted parameters are used as a sample of input–output of the neural network. Finally, the neural network will be trained by the samples of input–output.

To distinguish the measurements of different blocks at the decoder, there are two strategies for transmitting additional bit overhead to distinguish measurements of different blocks. The first strategy is to transfer the measurements block by block, and then add identifiers between the different block measurements to distinguish them.

Another strategy is to transmit the measurements in the order of sampling. The partial measurements of the partial sampling are first transmitted. The number of partial measurements of each block is $M_{0}$, so it is easy to identify at the decoder. Then, the decoder calculates the sampling rate of each block by the same neural network and formula as the encoder. In order to ensure the sampling rate calculated by the decoder to be consistent with the encoder, the input of the neural network must be the measurements after quantization and dequantization. Compared with the first strategy, the second strategy only needs to inform the corresponding decoder about the quantization parameters of the measurements of the two sampling processes, which requires very few additional bits overhead. When using 8 bits to quantize the measurements, the two strategies have little influence on the reconstruction through experiments. To quickly simulate and optimize models, we take the measurements without quantization as the input of the network in the following simulation experiment. In practical applications, the second strategy can be selected to transmit additional bit overhead.

We use the peak signal-to-noise ratio (PSNR) of the reconstructed image to measure the performance of the ABCS schemes. The higher PSNR indicates the better performance of the ABCS scheme. To facilitate the description, we use EFM-based, PFM-based, and LFM-based schemes to represent our proposed adaptive sampling methods based on exponential, polynomial, and logarithmic models, respectively.

### 6.1. Influence of the Number of Input Neurons on the Reconstruction Performance

**Figure 12:**
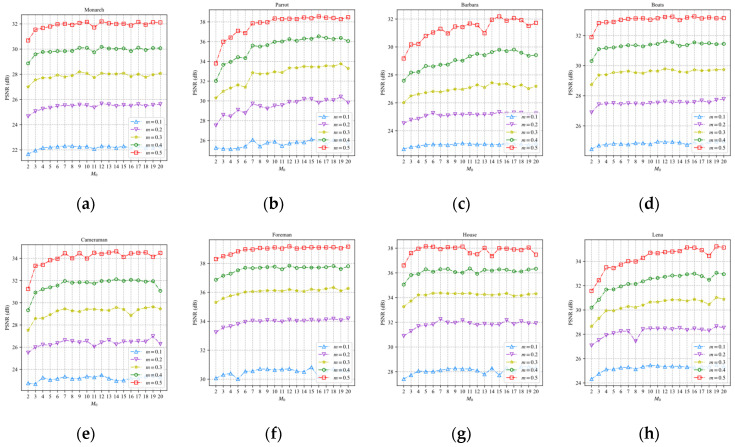
PSNR curves of eight test images: (**a**) Monarch; (**b**) Parrot; (**c**) Barbara; (**d**) Boats; (**e**) Cameraman; (**f**) Foreman; (**g**) House; (**h**) Lena.

We take the EFM-based scheme to analyze the influence of the number of input neurons on the reconstruction performance, where the parameter $a_{2}$ is fixed as $P_{0}=-2$. We obtain 19 neural networks for predicting model parameters by setting different numbers of $M_{0}$, where $M_{0}$ belongs to {2, 3, …, 20}. Each of the 19 neural networks was trained 20 times. The neural network with the least loss in 20 trained networks is used in the EFM-based scheme. Eight test images are sampled and reconstructed by the EFM-based scheme and BCS-SPL-DCT at different sampling rates. Figure 12 shows the PSNR curves of the eight reconstructed images for different numbers of $M_{0}$.

In Figure 12, the PSNR increases with the increase in $M_{0}$. The PSNR gradually slows down when $M_{0}$ is greater than 10. Therefore, we set $M_{0}$ to 10.

### 6.2. Influence of the Parameter Discretization on the Reconstruction Performance

**Figure 13:**
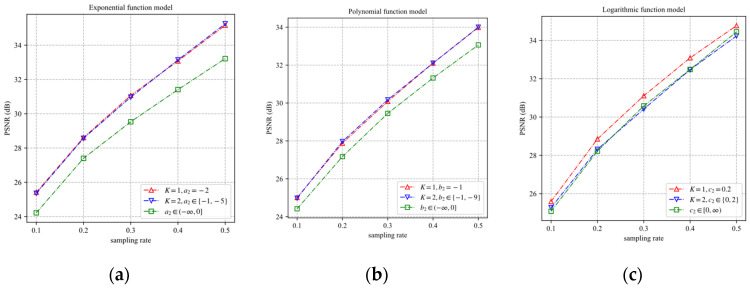
Average PSNR curves of the eight reconstructed images for the proposed ABCS schemes: (**a**) EFM-based scheme; (**b**) PFM-based scheme; (**c**) LFM-based scheme.

According to the discussion in Section 5.3, the more elements in the value set of parameters, the better the fitting performance of the distortion model. However, for the same neural network, the difficulty of predicting parameters will increase with the number of the parameters value sets. To find an optimal number of parameter value sets, we use the models of parameter discretization analyzed in Section 5.3 to sample the eight test images. There are nine models in total, and the neural network of each model was trained 20 times. Each neural network with the least loss in 20 trained networks is used for the ABCS scheme. The BCS-SPL-DCT is used to reconstruct the images from the sampled CS measurements. Figure 13 shows the average PSNR curves of the eight reconstructed images for the proposed ABCS schemes.

As shown in Figure 13, the EFM-based and PFM-based schemes with parameter discretization have significant PSNR gain. When the parameter $c_{2}$ is fixed as 0.2, the LFM-based scheme can obtain a more significant PSNR gain. These results verify the effectiveness of parameter discretization. Nevertheless, the LFM-based scheme cannot obtain a PSNR gain when the value set of the parameter $c_{2}$ has two values. This indicates that for the performance of the proposed ABCS schemes, the number of discrete values is not the more, the better. Compared with the fitting sets of $a_{2}$ and $b_{2}$ fixed to the one value, the EFM-based and PFM-based schemes do not have more PSNR gain when the fitting sets of $a_{2}$ and $b_{2}$ are fixed to the two values. Therefore, we fix the parameters $a_{2}=-2$, $b_{2}=-1$, and $c_{2}=0.2$ for the EFM-based, PFM-based, and LFM-based schemes, respectively.

### 6.3. Performance Comparison of Different ABCS Schemes

**Figure 14:**
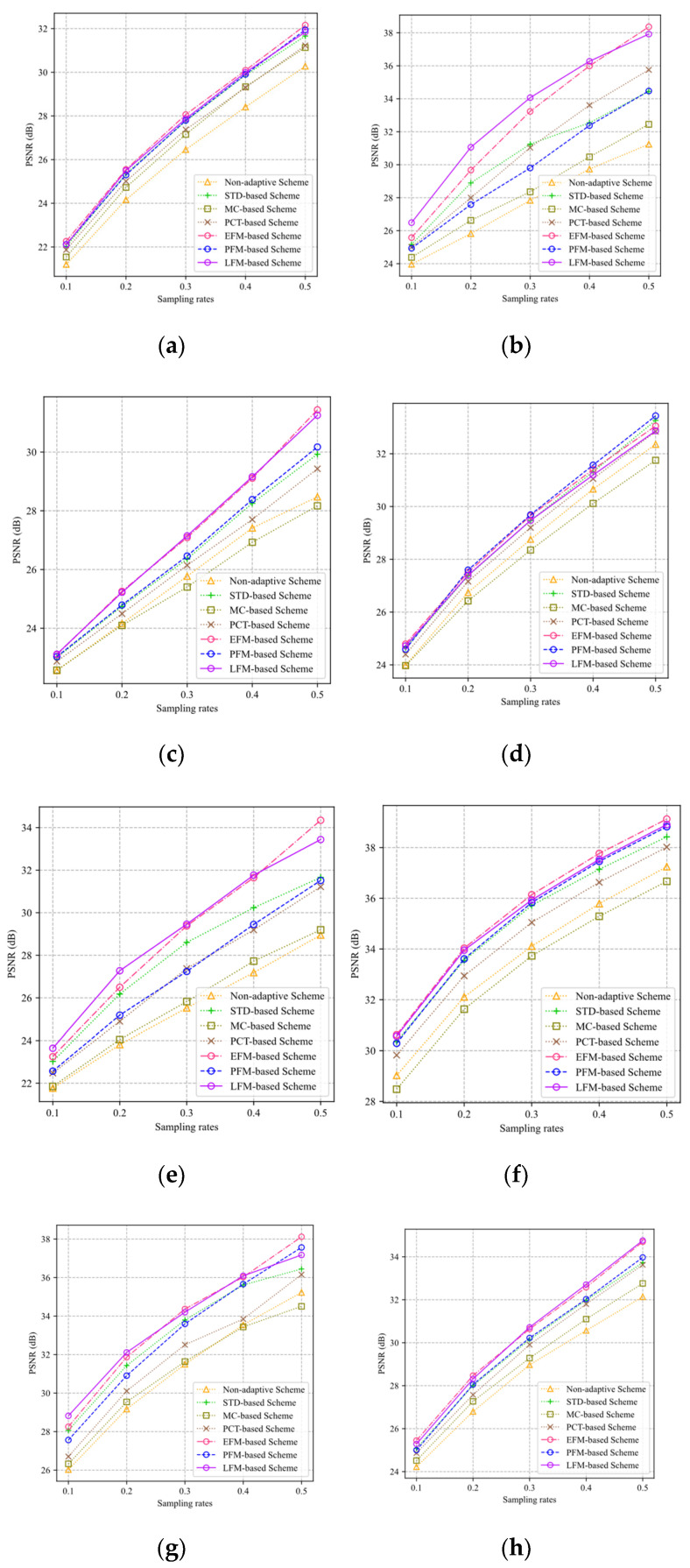
PSNR curves of eight test images: (**a**) Monarch; (**b**) Parrot; (**c**) Barbara; (**d**) Boats; (**e**) Cameraman; (**f**) Foreman; (**g**) House; (**h**) Lena.

In this section, the PSNRs of the reconstructed images are used to evaluate the performance of different ABCS schemes. The proposed ABCS schemes are compared with the ABCS scheme based on the standard deviation (STD-based) [19], the ABCS scheme based on the MC (MC-based) [10], and the ABCS scheme based on the PCT (PCT-based) [8]. Since Ref. [8] does not give an allocation strategy for a given sampling rate of image, we take Equation (8) in Ref. [8] as an allocation factor, and use Equation (4) to assign the sampling rate of each block. In our simulation, the parameters of the PCT-based scheme take $\theta_{\min}=0.4(B^{2}m_{\mathrm{goal}})$, $\theta_{\max}=8\theta_{\min}$, and the down-sampled image size is 64 × 64. Figure 14 shows the sampling rate–PSNR curves of eight test images for different ABCS schemes.

In Figure 14, the performance of the three proposed adaptive frameworks on eight test images is better than that of the non-adaptive scheme and the MC-based scheme. The EFM- and LFM-based schemes outperform other schemes at all sampling rates for Monarch, Parrot, Barbara, Cameraman, Foreman, House, and Lena. The EFM- and LFM-based schemes are slightly worse than the STD-based scheme at sampling rates of 0.4 and 0.5 for Boats. Especially for Parrot, Barbara, Cameraman, House, and Lena, the PSNR gains of the EFM- and LFM-based schemes are much higher than those of other schemes. The performance of the PFM-based scheme is not stable. The PFM-based scheme has better results than the STD-based scheme for Monarch, Barbara, Boats, Foreman, and Lena, but it has weaker results than the STD-based scheme for Parrot, Cameraman, and House. The PFM-based scheme has better results than the PCT-based scheme for all the test images except for Parrot.

**Figure 15:**
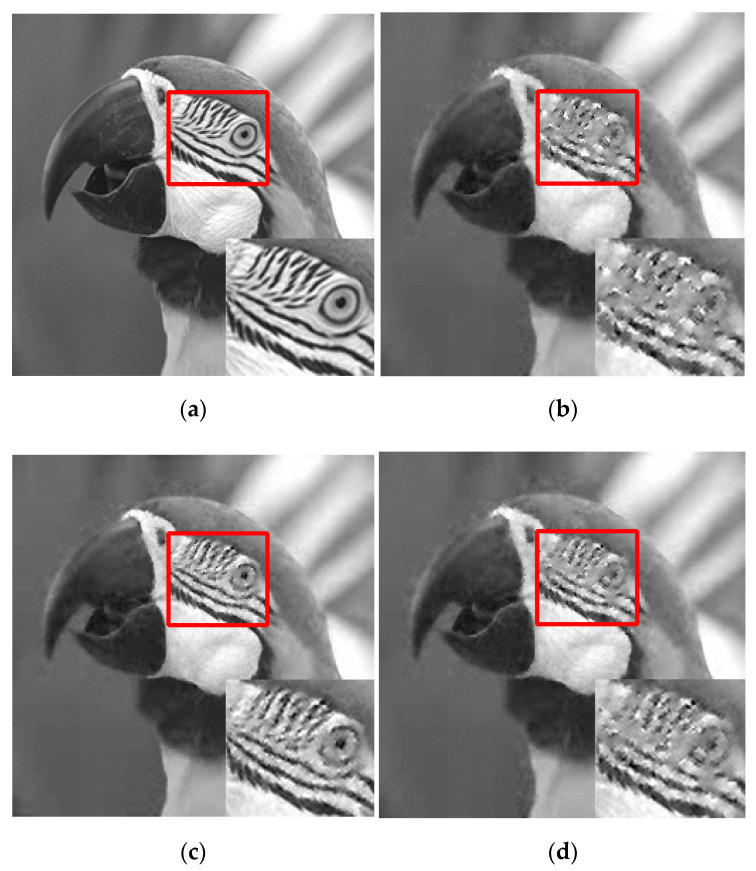

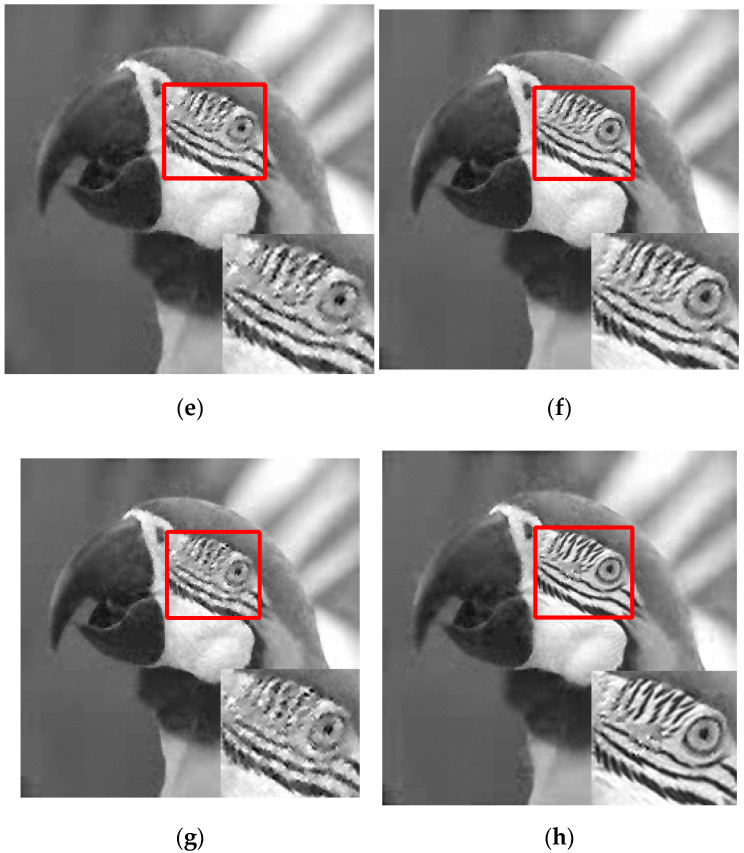
Visual comparisons of Parrot when using different ABCS schemes at sampling rate of 0.2: (**a**) original image; (**b**) Non-adaptive Scheme; (**c**) STD-based scheme; (**d**) MC-based scheme; (**e**) PCT-based scheme; (**f**) EFM-based scheme; (**g**) PFM-based scheme; (**h**) LFM-based scheme.

Figure 15 shows the visual reconstruction results of Parrot through different ABCS schemes at a sampling rate of 0.2, where the local regions of the reconstructed images are enlarged to highlight the differences. For the non-adaptive scheme, the enlarged region loses many texture details, which makes us unable to distinguish the eye of parrot. Compared with the non-adaptive scheme, the ABCS schemes have better visual effects. Among the ABCS schemes, the LFM-based scheme has the best visual effect, followed by the EFM-based, STD-based, PCT-based, PFM-based, and MC-based schemes.

**Figure 16:**
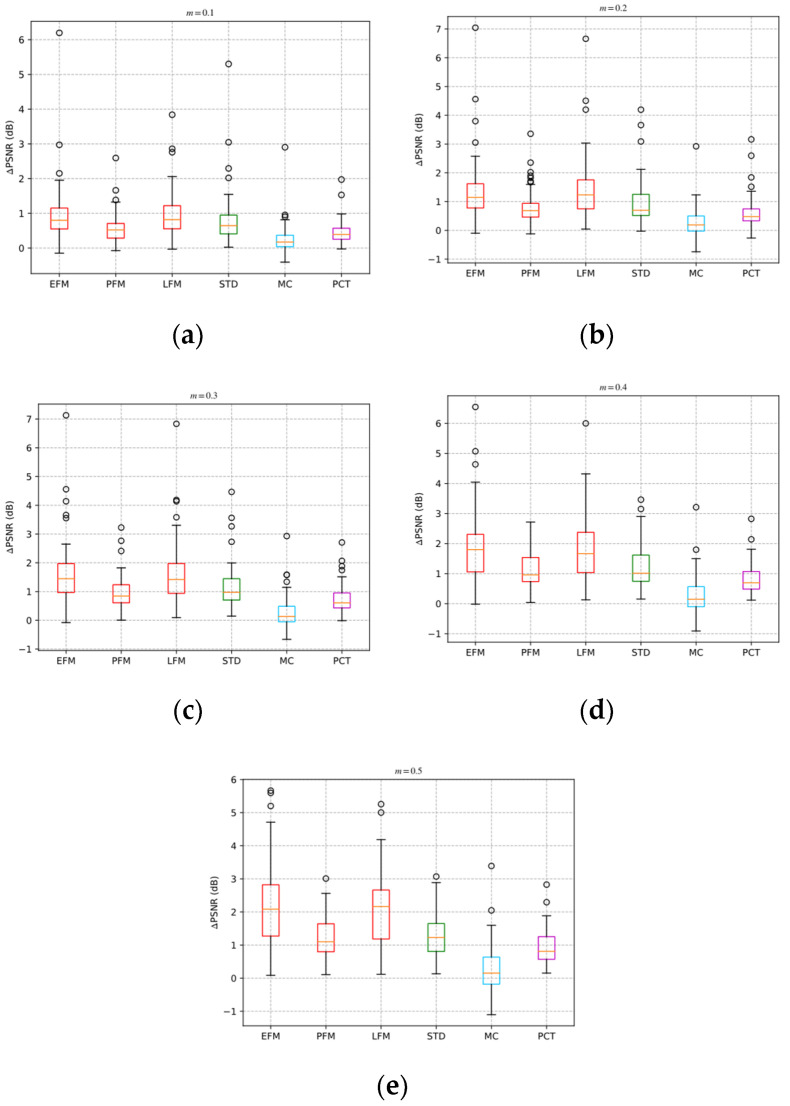
PSNR gains of different ABCS schemes for different sampling rates: (**a**) m = 0.1; (**b**) m = 0.2; (**c**) m = 0.3; (**d**) m = 0.4; (**e**) m = 0.5.

In addition, we compare the performance of different ABCS schemes on the BSD68 dataset. Table 3 shows the average PSNR values of the BSD68 dataset for different ABCS schemes. Figure 16 shows the boxplots of PSNR gains of the BSD68 dataset for different ABCS schemes.

In Table 3, ∆PSNR represents the PSNR gain brought by the adaptive sampling framework compared to BCS. The average PSNRs of all adaptive ABCS schemes are higher than non-adaptive schemes. Among the STD-based, MC-based, and PCT-based schemes, the average PSNR of the STD-based scheme is the highest. At sampling rates of 0.1, 0.2, 0.3, 0.4, and 0.5, the average PSNR value of the EFM-based scheme is higher than that of the STD-based scheme by 0.14 dB, 0.4 dB, 0.46 dB, 0.66 dB, and 0.9 dB, respectively. The average PSNR of the PFM-based scheme is higher than that of the STD-based scheme by −0.24 dB, −0.15 dB, −0.17 dB, −0.06 dB, and −0.02 dB, respectively. The average PSNR value of the LFM-based scheme is higher than that of the STD-based scheme by 0.16 dB, 0.45 dB, 0.48 dB, 0.63 dB, and 0.77 dB, respectively.

In Figure 16, each boxplot contains five horizontal lines, which represent the minimum, lower quartile, median, upper quartile, and maximum of the dataset of ∆PSNR from bottom to top. The box is drawn from the lower quartile line to the upper quartile line. The small circles represent the outliers of ∆PSNR. The lower quartile, median, and maximum of the PSNR gain of the EFM-based and LFM-based schemes are obviously better than other schemes. The lower quartile, median, and maximum of the PFM-based scheme are worse than those of the EFM-based, LFM-based, and STD-based schemes.

The minimum of the PSNR gain of the EFM-based and LFM-based schemes is less than 0 at the sampling rates of 0.1, 0.2, and 0.3. This is because the EFM-based and LFM-based schemes do not improve the reconstruction performance of some images with high block similarity. The minimum of the PSNR gains is −0.15 dB, indicating a limited adverse effect on the reconstruction performance of such images.

It can be found from Table 3 and Figure 16 that the LFM-based scheme has the best performance at the sampling rates of 0.1, 0.2, and 0.3. The EFM-based scheme has the best performance at the sampling rates of 0.4 and 0.5. The PFM-based scheme is slightly worse than the STD-based scheme but better than the MC-based and PCT-based schemes.

### 6.4. Complexity Analysis

The additional computational complexity of the proposed ABCS scheme mainly comes from the model parameters prediction, the estimation of λ or $\log_{2}(\lambda )$, and sampling rate calculation.

The distortion model parameter prediction of a block based on the neural network requires about $6M_{0}+6\times 4+4\times 3=96$ multiplications and 96 additions. 

The estimation of λ or $\log_{2}(\lambda )$ is applied to all image blocks, so each block needs at most four multiplications and four additions, according to Equations (16), (19) and (22).

For sampling rate estimation, the EFM-based scheme requires one logarithm, two multiplications, and one addition, while the PFM-based or LFM-based scheme requires one addition and one multiplication. Among the three models, the EFM-based scheme requires an additional logarithmic calculation, which has the highest complexity. If the logarithmic calculation is estimated by using seven series expansion, it takes about 26 multiplications and six additions. The additional amounts of calculations of the EFM-based scheme will not exceed half of that of the variance.

Compared with the calculation of sampling the CS measurements at a sampling rate of 0.1, the additional amounts of computation of the EFM-based scheme will not exceed 1.9%, and those of the PFM- and LFM-based schemes will not exceed 1.5%. Moreover, with the sampling rate, the proportion of additional amounts of calculations is further reduced.

## 7. Conclusions

Here, we propose a low-complexity adaptive sampling method for BCS. The exponential, polynomial, and logarithmic functions are used to describe the mapping relationship between the sampling rate and the distortion of the reconstructed image block. The block sampling rate is quickly estimated based on distortion minimization. The model parameters can be predicted by the simple neural network. To improve the learning ability of the neural network, we discretize the fitting interval of one of the parameters to reduce the diversity of the training samples. Although the proposed ABCS schemes need substantial calculation to collect the training samples of the neural network, this is acceptable because it is off-line. Experiments show that the proposed method is effective and has better performance than other popular methods.

## Figures and Tables

**Table 1:** The number of operations required for different features.

Features	Expression	Addition	Multiplication	Other
CS sampling	$y_{i}=\Phi_{M_{i}}x_{i}$	$M_{i}(B^{2}-1)$	$M_{i}B^{2}$	-
2D transform	$T_{i}=FX_{i}F^{T}$	$2B^{2}(B-1)$	$2B^{3}$	-
PCT [8]	$\begin{array}{l} P=\mathrm{sign}(C(X_{d}))\\ F=\mathrm{abs}\left(C^{-1}(P)\right)\\ Saliency\;Map=G*F^{2} \end{array}$	$\frac{5I_{d}^{3}+3I_{d}^{2}}{N}$	$\frac{5I_{d}^{3}+9I_{d}^{2}}{N}$	-
Variance [16,19]	$\sigma_{i}^{2}=\frac{1}{B^{2}}\sum_{j=0}^{B^{2}}{\left(x_{i,j}-{\bar{x}}_{i}\right)}^{2}$	$3B^{2}-2$	$B^{2}+2$	-
Spatial entropy [20]	$H_{i}=-\sum_{j=0}^{255}p_{j}\log(p_{j})$	255	256	$p_{j}$ $,\log(p_{j})$
Measurement contrast (MC) [10]	$w_{i}=\sum_{j=1}^{N^{2}/B^{2}}{\left\|y_{init,i}-y_{init,j}\right\|}_{2}^{2}$	$(0.6B^{2}-1)N$	$0.3B^{2}N$	-
Sensing entropy [13]	$\begin{array}{c} d_{i,j}={\left\|y_{i}-y_{i\circ j}\right\|}_{2}\\ H_{\mathrm{inter},i}=\frac{\min\left\{d_{i,j}|j=1,2,\cdots ,8\right\}}{\max\left\{d_{i,j}|j=1,2,\cdots ,8\right\}}\\ H_{\mathrm{intra},i}=1/2+\mathrm{sgn}(y_{i,1})\frac{y_{i}^{T}\varphi_{b}}{{\left\|y_{i}\right\|}_{2}{\left\|\varphi_{b}\right\|}_{2}}\\ H_{t}=\beta H_{\mathrm{intra},i}+(1-\beta )H_{\mathrm{inter},i} \end{array}$	$19B^{2}-11$	$11B^{2}+4$	min{·},max{·},

**Table 2:** The PCCs between different vectors of $G_{1}(a_{2})$.

	*a*_2_	−0.01	−1	−2	−3	−4	−5	−6	−7	−8	−9
*a*_2_											
−0.01		1	0.998	0.991	0.980	0.965	0.948	0.930	0.910	0.890	0.870
−1		-	1	0.998	0.991	0.981	0.967	0.952	0.935	0.918	0.900
−2		-	-	1.000	0.998	0.992	0.982	0.970	0.957	0.942	0.927
−3		-	-	-	1	0.998	0.992	0.984	0.974	0.962	0.949
−4		-	-	-	-	1	0.998	0.993	0.986	0.977	0.966
−5		-	-	-	-	-	1	0.998	0.994	0.988	0.980
−6		-	-	-	-	-	-	1	0.999	0.995	0.989
−7		-	-	-	-	-	-	-	1	0.999	0.996
−8		-	-	-	-	-	-	-	-	1	0.999
−9		-	-	-	-	-	-	-	-	-	1

**Table 3:** Average PSNR (dB) of BSD68 datasets for different ABCS schemes.

Sampling Scheme		Sampling Rate				
		0.1	0.2	0.3	0.4	0.5
Non-Adaptive Scheme	PSNR	22.89	24.69	26.17	27.55	28.90
STD-Based Scheme	PSNR	23.70	25.65	27.32	28.74	30.17
	∆PSNR	0.81	0.96	1.14	1.19	1.27
MC-Based Scheme	PSNR	23.14	24.97	26.44	27.82	29.16
	∆PSNR	0.25	0.28	0.27	0.27	0.26
PCT-Based Scheme	PSNR	23.33	25.30	26.90	28.39	29.86
	∆PSNR	0.44	0.61	0.73	0.84	0.95
EFM-Based Scheme	PSNR	23.84	26.05	27.78	29.40	31.07
	∆PSNR	0.95	1.36	1.61	1.85	2.17
PFM-Based Scheme	PSNR	23.46	25.50	27.15	28.68	30.15
	∆PSNR	0.57	0.81	0.98	1.13	1.25
LPM-Based Scheme	PSNR	23.86	26.10	27.80	29.37	30.94
	∆PSNR	0.97	1.41	1.63	1.82	2.04

## Data Availability

Not applicable.
